# Strategies Using Gelatin Microparticles for Regenerative Therapy and Drug Screening Applications

**DOI:** 10.3390/molecules26226795

**Published:** 2021-11-10

**Authors:** Teruki Nii

**Affiliations:** 1 Graduate School of Systems Life Sciences, Kyushu University, 744 Motooka, Nishi-ku, Fukuoka 819-0395, Japan; nii.teruki.204@m.kyushu-u.ac.jp; 2 Department of Applied Chemistry, Faculty of Engineering, Kyushu University, 744 Motooka, Nishi-ku, Fukuoka 819-0395, Japan

**Keywords:** biotechnology, drug delivery, drug research model, gelatin, regenerative medicine

## Abstract

Gelatin, a denatured form of collagen, is an attractive biomaterial for biotechnology. In particular, gelatin particles have been noted due to their attractive properties as drug carriers. The drug release from gelatin particles can be easily controlled by the crosslinking degree of gelatin molecule, responding to the purpose of the research. The gelatin particles capable of drug release are effective in wound healing, drug screening models. For example, a sustained release of growth factors for tissue regeneration at the injured sites can heal a wound. In the case of the drug screening model, a tissue-like model composed of cells with high activity by the sustained release of drug or growth factor provides reliable results of drug effects. Gelatin particles are effective in drug delivery and the culture of spheroids or cell sheets because the particles prevent hypoxia-derived cell death. This review introduces recent research on gelatin microparticles-based strategies for regenerative therapy and drug screening models.

## 1. Introduction

As representative biomaterials, chitosan [1,2], alginate [3,4], hyaluronic acid [5,6], collagen [7,8], gelatin [9,10], polylactic acid [11], polyglycolic acid [12,13], poly (lactic-*co*-glycolic acid) [14,15,16], or polyethylene glycol [17,18] are well known. Among the biomaterials, gelatin is often used for medical [19,20] or cosmetics [21] because gelatin is water-soluble [22], low inflammatory [23], and promotes high cell adhesion [24]. Gelatin formulation, such as a scaffold, has been investigated for cell transplantation [25,26,27]. Moreover, it has been reported that gelatin fiber supports the culture of cell sheets [28,29]. In addition to these non-spherical shape types, gelatin particles, especially micro size, have been investigated in the field of in vivo therapy or in vitro cell culture. This paper is a short review of recent research on gelatin microparticles-based biotechnology strategies for regenerative therapy and drug screening.

## 2. Protocol for the Preparation of Gelatin Microparticles 

An aqueous gelatin solution is added to the olive oil by stirring for 10 min at 40 °C to prepare the water-in-oil emulsion. The emulsion temperature is decreased at 4 °C for the natural gelation of gelatin solution to obtain non-crosslinked hydrogel microspheres. The resulting gelatin microparticles (GMs) are washed a few times with cold acetone to exclude the residual oil completely. Next, GMs are fractionated by appropriate size using sieves [30]. Note that it is better to perform this protocol on ice because the non-crosslinked GMs are easily degraded at room temperature. 

## 3. Crosslinking Methods

Non-crosslinked GMs cannot be used in cell culture or animal experiments because of the quick degradation. To obtain the formulation with appropriate degradation, chemical or dehydrothermal crosslinking processes are needed. The comparison of the two methods is shown in Table 1.

Among the chemical crosslinking reagents, it has been reported that there are some differences. For example, when the cells were cultured on the gelatin formulations crosslinked by genipin, cell seeding efficiency was significantly lower than aldehyde or carbodiimide. In addition, when the carbodiimide was used for crosslinking reagent, the gelatin formulations presented poor anti-hydrolysis ability [40]. Due to the reports, the aldehyde is often selected for crosslinking. Recently, dehydrothermal crosslinking has been noted because of the ease of handling [23]. If the machine for vacuum heating can be obtained, dehydrothermal crosslinking is the most appropriate choice.

## 4. Gelatin-Based Drug Delivery Systems

**Figure 1:**
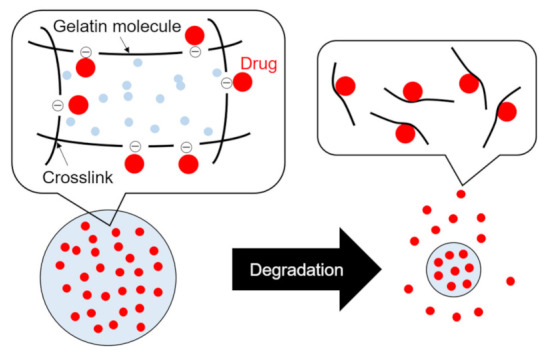
A schematic representation of drug release from gelatin particles (when the isoelectric point of gelatin is negative.). The gelatin used for sustained drug release can be selected considering the isoelectric point of the drug (If the drug to be released is basic, gelatin with a negative charge is preferable.). Drugs and gelatin molecules interact by physicochemical interaction (e.g., ionic or hydrogen interaction). When the gelatin particles are degraded, the drugs with gelatin molecule debris are rapidly released with time.

Growth factors are needed to enhance cell activity or function [41,42,43]. Therefore, the delivery of growth factors to cells would be a promising strategy for treating diseases. However, growth factors are quickly degraded, so the carrier for growth factors contained is essential. Gelatin molecules can interact with growth factors by electronic interaction because gelatin is a denatured form of collagen, a major extracellular matrix (ECM) component [44]. When the collagenase degrades the gelatin particles, the growth factors are released with gelatin molecule debris (Figure 1) [44,45]. This drug release mechanism is effective in tissue regeneration. When the gelatin particles containing growth factors are injected into the damaged tissues, growth factors are rapidly released, leading to tissue regeneration. This is due to the high secretion level of collagenase (e.g., vascular endothelial growth factor or matrix metalloproteinase) in the damaged tissues. In addition, the release speed of growth factors can be controlled by changing the crosslinking degree of gelatin molecules [46,47]. For example, when gelatin particles with the slow release of growth factors are needed, you should introduce a higher concentration of crosslinking reagents or a longer time for dehydrothermal crosslinking. Taken together, the mechanism of matrix-degradation-based drug release characterization is one of the attractive properties of gelatin [22,44].

## 5. Applications of Gelatin Microparticles

In regenerative therapy and drug research models, enhanced cell activity or function is one of the most important concepts [48]. To achieve regenerative therapy, cells in the damaged tissue should proliferate by obtaining high cell activity. In the case of drug screening models, the cell activity or function of models should be close to that of natural tissues. To assist the enhancement of cell activity or function, GMs are often used. In this chapter, regenerative therapy and drug research model using GMs are introduced.

### 5.1. Regenerative Therapy

Table 2 summarizes some recent reports on regenerative therapy using gelatin microparticles.

There are two important factors for the achievement of tissue regeneration using materials transplantation into the damaged tissues. One is the speed of material degradation. To regenerate the tissue damaged, cells should actively migrate and proliferate in the defective site. Therefore, the speed of cell migration and material degradation should be linked and synchronized [22]. As mentioned above, the degradation profile of gelatin particles can be easily modified by the crosslinking reagent concentration or the dehydrothermal crosslink period. Therefore, gelatin particles are suitable for tissue regeneration in terms of degradation control. The second is the disappearance of the material. The remaining materials are unnecessary after the tissue regeneration is completed. Even though wound healing and tissue regeneration are achieved, the permanent existence of materials would induce inflammation [58]. Gelatin particles are materials capable of solving this problem because they are degraded into harmless amino acids to the body. 

### 5.2. Drug Research Model

Table 3 summarizes the research on the GMs-based spheroids for drug research.

Drug discovery is one of the most promising strategies to treat intractable diseases. Several hard processes should be passed to develop new drugs: drug screening using cells, preclinical study, and clinical study [68]. However, the drug efficacy of drug screening is often different from that of a preclinical or clinical study, leading to drug development failure [69,70]. This is mainly due to the difference in environmental conditions between in vitro and in vivo [71,72]. Cells are usually cultured by a two-dimensional culture system of a dish or plate. However, cells in the body environment tend to interact with each other in a three-dimensional (3D) manner. The interaction leads to an enhanced cell function, such as proliferation [73,74], differentiation [75,76], or metabolism [77]. Based on the characteristics, 3D tissue-like models, such as spheroids [78,79,80,81,82], organoids [83,84,85,86], or microfluidics systems [87,88,89], have been recently demonstrated. However, hypoxia is induced in the center of spheroids, leading to cell death [90,91]. Due to cell death, it is difficult to culture the spheroids for a long period to investigate the cell function. GMs have been incorporated into the spheroids to tackle the issues because oxygen or nutrients can be permeated through the water phase of gelatin gels [30]. The function of spheroids incorporating GMs is higher than that without GMs incorporation [23,30]. For example, when the insulinoma spheroids are prepared, the insulin secretion is enhanced. The model is useful as a tool for type 1 diabetes drug research [64]. 

In addition, the drug delivery system technology of GMs is effective in the drug research model. To enhance the cell function in vitro, similar to in vivo, the release of drugs, which enhance the cell function or activity, is important. Based on this reason, spheroids incorporating GMs containing drugs have been demonstrated for the anti-cancer drug research model [39,66,67]. Under the tumor environment, cancer cells interact with cancer cells and stromal cells of cancer-associated fibroblasts (CAF) [92,93]. Because CAF are always activated in vivo, it is important to activate CAF in vitro to mimic the tumor environment [94]. Therefore, to enhance and activate the CAF, CAF spheroids incorporating GMs containing drugs have been prepared. In addition, when the activated CAF spheroids and cancer cells are co-cultured via model basement membrane, cancer cells are effectively migrated with the penetration through the membrane. This CAF spheroids/cancer cells co-culture model is a promising tool to evaluate the invasion ability of cancer cells in vitro; therefore, the effect of candidate anti-invasion drugs can be investigated using the model [39,66].

## 6. Future Perspective and Conclusions

Biomaterial usage for in vivo therapy or in vitro research has been noted because the biomaterial enables the enhancement of cell potentials, such as proliferation, differentiation, or metabolism. For further development of the field, it is essential to use material of low inflammatory induction. Because gelatin is a denatured form of collagen, a major component of proteins, gelatin is a suitable material for patient-friendly therapy. In addition, gelatin can support cell viability by providing collagen proteins to the cells. However, ECM components consist not only of collagen but also polysaccharides [95]. Based on this cell characteristic, polysaccharides-based biomaterials, such as alginate, chitosan, or hyaluronic acid, are also essential to enhance cell activity or function. Therefore, the combination of polysaccharides-based biomaterials and gelatin materials would further develop regenerative therapy or drug research models. 

In this review, regenerative therapy and drug research models using gelatin microparticles (GMs) are introduced. In both two applications, collagenase-triggered drug release is the common keyword. In the case of regenerative therapy, the higher secretion of collagenase in the injured site is utilized. Because the drug is released from GMs only on injured sites, it is possible to enhance the drug effects or reduce the side effects. When the GMs are incorporated into the spheroids for drug research models, collagenase secretion by the 3D cell-cell interaction can enhance the drug release. This on-off drug release would also be effective in other applications in the future, such as vaccines. The allergen must be administered to antigen-presenting cells (APC), such as dendritic cells. When the allergen is diffused, severe anaphylaxis will occur. Therefore, to achieve efficient vaccines, allergen should be intensively administered to APC. To tackle this issue, GMs-based allergen release would be promising. Because the sites of allergen administration are healthy, the allergen is not leaked from gelatin microparticles after the injection. After the GMs are selectively up taken into the APC by the APC-specific ligand coating, the allergen is released from GMs “inside” the APC. This is because the collagenase exists as the intracellular enzyme. Therefore, GMs are attractive drug carriers for many applications.

## Figures and Tables

**Table 1:** Comparison of features between chemical and dehydrothermal crosslinking methods.

Points Compared	Crosslinking Method	
	Chemical	Dehydrothermal
Instrument needed	Nothing	Oven
Temperature (°C)	40	140~160
Particle condition under process	Liquid	solid
Crosslinking reagent added	Aldehyde, isocyanates, acyl azides, or carbodiimide [31,32,33,34]	Nothing
Stop reagent added	Glycine [35]	Nothing
Time required (days)	1	2~5
Merit	Safety condition (room temperature condition) [36] Particular instrument is not needed [37].	Easy to handle [38] Aggregation is not formed because of the solid condition.
Demerit	Aggregation is sometimes formed.	Particular instrument is needed [39].

**Table 2:** Examples of regenerative therapy and tissue regeneration strategies using gelatin microparticles.

Ref.	Date	Tissue Regenerated	In Vitro (Cell Type)/In Vivo (Animal Type)	Growth Factors Released	Main Results
[49]	2015	Cardiac	In vitro (human cardiac cells derived from iPS cells)/In vivo (mouse)	-	The survival rate of stacked cell sheets was improved by incorporating gelatin microparticles between each cell sheet.
[50]	2017	Blood vessels	In vitro (human umbilical vein endothelial cells and human dermal fibroblast cells)/In vivo (mouse)	Platelet-rich plasm A(PRP)	Gelatin microparticles containing PRP promoted the formation of capillaries and microvascular networks.
[51]	2018	Sternal	In vivo (rabbit)	PRP	PRP-gelatin microparticles injection showed a significantly higher indicator of sternal healing than only gelatin microparticles injection.
[52]	2018	Bone	In vitro (mouse mesenchymal stem cells and mouse macrophages)	Bone morphogenic protein-2 (BMP-2)	The gelatin microparticles were prepared to be preferentially degraded by pro-inflammatory macrophages, leading to the spatiotemporal BMP-2 release. The strategy enabled to achieve the efficient bone differentiation of stem cells.
[53]	2018	Cardiac	In vivo (rat)	Basic fibroblast growth factor (bFGF)	Gelatin microparticles capable of bFGF control release showed the improvement of cell sheets’ viability.
[54]	2019	Cartilage	In vitro (human periosteum derived cells)	Transforming growth factor-β1 (TGF-β1)	TGF-β1 release from gelatin microparticles promotes the chondrogenic differentiation of human periosteum-derived cells.
[55]	2019	Bone	In vitro (rabbit mesenchymal stem cells)/In vivo (rabbit)	BMP-2	BMP-2 release system of gelatin microparticles is effective in bone regeneration of X-ray-radius defects.
[56]	2021	Cartilage and disk	In vitro (human stem cells)/In vivo (rat)	Matrilin3 and TGF-β3	Chondrogenic differentiation was promoted when gelatin particles containing Matrilin-3 and TGF-β3 were incorporated into stem cell spheroids while preventing hypertrophy.
[57]	2021	Masseter muscle	In vitro (rat stem cells)	bFGF and PRP	The combination of cell transplantation and the drug release system efficiently differentiated stem cells towards muscle lineage.

**Table 3:** In vitro drug research studies using 3D cell/tissue spheroids combined with gelatin microparticles.

Ref.	Date	Tissue or Disease	Cells Used	Growth Factors or Drugs Released	Main Results
[59]	2017	Epithelial	Mammary epithelial cells	-	β-casein expression of epithelial spheroids incorporating gelatin microparticles coated with Matrigel was higher than microparticles-free spheroids.
[60]	2017	Cancer	Cancer-associated fibroblasts and cancer cells	-	Cancer cells and cancer-associated fibroblasts (CAF) spheroids combined with gelatin particles showed a stromal matrix rich in collagen deposition and expressed the desmoplastic reaction markers.
[61]	2017	Epithelial	Mammary epithelial cells and preadipocyte cells	-	Epithelial-preadipocytes multicellular spheroids incorporating gelatin microparticles showed the enhancement of β-casein expression compared to spheroids in the absence of the gelatin microparticles.
[62]	2017	Bone	Pre-osteoblast cells	Bone morphogenic proteins-2 (BMP-2)	When spheroids incorporating gelatin microparticles containing BMP-2 were prepared, efficient osteogenic differentiation was observed compared to spheroids incorporating gelatin microparticles.
[63]	2018	Cancer	Cancer-associated fibroblasts and cancer cells	-	Cancer cells and CAF spheroids embedded gelatin particles enabled the evaluation of the anti-cancer drug effects efficiently.
[64]	2018	Pancreas	Insulinoma cells	-	The insulinoma spheroids incorporating gelatin microparticles prompted the secretion of insulin.
[65]	2018	Cancer	Cancer cells, endothelial cells, and fibroblasts	-	3D tissue model consisting of cancer cells, endothelial cells, and fibroblasts was prepared. In this model, aberrant capillary-like structures were observed, which are important events of breast cancer progression.
[39]	2019	Cancer	Cancer-associated fibroblasts and cancer cells	p53 inhibitor	CAF spheroids incorporating gelatin microparticles containing a p53 inhibitor were prepared to activate the CAF function in vitro, similar to in vivo. The activated CAF spheroids can promote the invasion ability of cancer cells.
[66]	2020	Cancer	Cancer-associated fibroblasts and cancer cells	Transforming growth factor-β (TGF-β)	CAF spheroids incorporating gelatin microparticles containing TGF-β enabled increased invasion rate of cancer cells, responding to TGF-β concentration.
[67]	2020	Cancer	Cancer-associated fibroblasts, macrophages, and cancer cells	Adenosine and TGF-β	3D tumor-associated macrophages incorporating gelatin microparticles containing adenosine and 3D CAF incorporating gelatin microparticles containing TGF-β were combined. This system can mimic the tumor microenvironment, responding to the tissue region.
